# The complete mitochondrial genome of blue pansy, *Junonia orithya* (Lepidoptera: Nymphalidae: Nymphalinae) from Pakistan

**DOI:** 10.1080/23802359.2020.1768912

**Published:** 2020-05-27

**Authors:** Saira Bibi, Muhammad Fiaz Khan, Aqsa Rehman, Muzafar Shah, Faisal Nouroz, Aneesa Nayab

**Affiliations:** a Department of Zoology, Hazara University, Mansehra, KPK, Pakistan; b Women University Swabi, >Pakistan; c Department of Zoology, University of Swat, Saidu Sharif, Pakistan; d Department of Bioinformatics, Hazara University, Mansehra, Pakistan; e Department of Botany, Abdul Wali Khan University, Mardan, Pakistan

**Keywords:** Blue pansy, complete, mitochondrial genome, phylogenetic analysis, Pakistan

## Abstract

*Junonia orithya’s* complete mitochondrial genome (mitogenome) is determined to be 14,214 bp in length, including 37 typical mitochondrial genes and an AT-rich region. Its gene order and orientation are identical to those of other butterfly species. All PCGs are initiated by typical ATN codons, except for *cox1* gene which is started by CGA codon. Nine genes use complete termination codon (TAA), whereas the COX*1, COX2*, NADH*1* and NAH*4* genes end with single T. Except for trnS1(AGN), all tRNA genes display typical secondary cloverleaf structures as those of other insects. The 331 bp long AT-rich region contains several features common to the other lepidopterans, such as the ATAGA motif followed by a 18 bp poly-T stretch, two microsatellite-like (TA) 9 elements, a 5 bp poly-A stretch immediately upstream of trNAM gene from Pakistan.

Because of its simple structure, abundant distribution, maternal inheritance, mitochondrial genome, and its high mutation rate is thought to be the ideal marker for the genetic diversity in population studies, species identification, and molecular phylogeny (Dormann et al. 2008; Bibi and Fiaz Khan 2019). In the form of mitochondrial DNA, genetic information offers a base to manage and protect the diversity of biology and for the interpretation of evolutionary accounts of varied biological species it allows the researchers (Bernt et al. 2013). Most of the animals’ self-replicating mitochondrial DNA is about 16-kb long; circular DNA molecule encodes for 13 protein-coding genes, (*COI–III, Cytb b, ND1–6* and *4L*, *ATPase* 6 and *ATPase* 8), 22 tRNAs, 2 rRNAs (*12S, 16S*), also having control region that controls its replication and transcription, the gene content and organization of mitochondrial DNA is quite conserved, this conserved characteristics facilitates their identification and placement (Uzoma et al. 2011). Five hundred species are there in Nymphalinae (Lepidoptera: Nymphalidae), which is distributed nearly all around the world (Harvey and Pagel 1991). Though the systematics and taxonomy of Nymphalinae still standup as a provocative issue and waiting for further investigations (Shi et al. 2015). Now, only three complete mitogenomes of Nymphalinae have been reported, including *Melitaea cinxia* (Melitaeini), *Kallima inachus* (Kallimini) and blue pansy (Pawar and Deshpande 2016). As in Pakistan, this species is in abundance, no study reported so far on the complete mitochondrial genome of blue pansy, namely, *Junonia orithya*, and preliminarily associated its sequence to other Nymphalidae mitogenomes, in order to deliver the taxonomic and (Kim et al. 2011) phylogenetic studies of Nymphalid butterflies for more useful information.

*Junonia orithya’s* complete mitogenome is a circular moleculeof 15,214 bp in length, typically containing 13 protein-coding genes (PCGs), 2 ribosomal RNA genes, 22 transfer RNA genes (tRNAs), and 1 major non-coding AT-rich region. Its gene order and alignment are identical to those of the other butterflies, 15 intergenic spacers (155 bp in total) and 11 overlapping regions (30 bp in total). Besides, the AT-rich region are detached throughout the whole genome. Toward AT (80.4%), the nucleotide compositions are suggestively biased which is well within the range of other sequenced Nymphalids, from 79.1% in *Eumenis autonoe* (Kim et al. 2011) to 81.9% in *Parathyma sulpitia*. The mitogenome nucleotide skewness (GC-skew ¼ 0.184, AT-skew ¼ 0.017,) by a typical ATN codon, all PCGs are initiated, except for the gene COX*1*, which uses as start codon unusual CGA (R) as observed in most of the other sequenced nymphalids (Hwang et al. 2013). Stop codon (TAA) used by nine PCGs, other four genes end with a single nucleotide T. Typical predicted secondary cloverleaf structures except for *trnS1* (AGN), contain all tRNAs whose dihydrouridine (DHU) arm is replaced by a simple loop, as seen in all other determined nymphalids (Catchen et al. 2013). Furthermore, the two rRNAs (775 bp rrnS and 1326 bp rrnL) are also pointedly biased toward AT nucleotides (82.7% for rrnL and 84.9% for rrnS). The 331 bp AT-rich region exhibits the highest AT content (94.9%) and contains several structures characteristic of lepidopterans, such as the ATAGA motif followed by a 18 bp poly-T stretch, two microsatellite-like (TA) 9 elements, a 5 bppoly-A stretch presented immediately upstream of *trnM* gene. Moreover, presence of a duplicated 38-bp repeated element, one of the famous features of the AT-rich region is the similar case that has been detected in the nymphalid species, *E. autonoe*, which harbors 10 identical 27 bp long tandem repeats and one 13 bp long incomplete repeat (Kim et al. 2011).

Collected from village Chhajjian, having gps coordinates 33.8832° N, 73.0333° E Voucher specimens (JO2132) were fixed in 10% formalin and transported to Department of Zoology, Hazara University, Mansehra, Pakistan where it is stored in the museum. We retrieved the complete mitochondrial genome sequence by blast search in NCBI in which the species show 100% bootstrap replicates except an out group showed 96% as shown in Figure 1.

**Figure 1.**
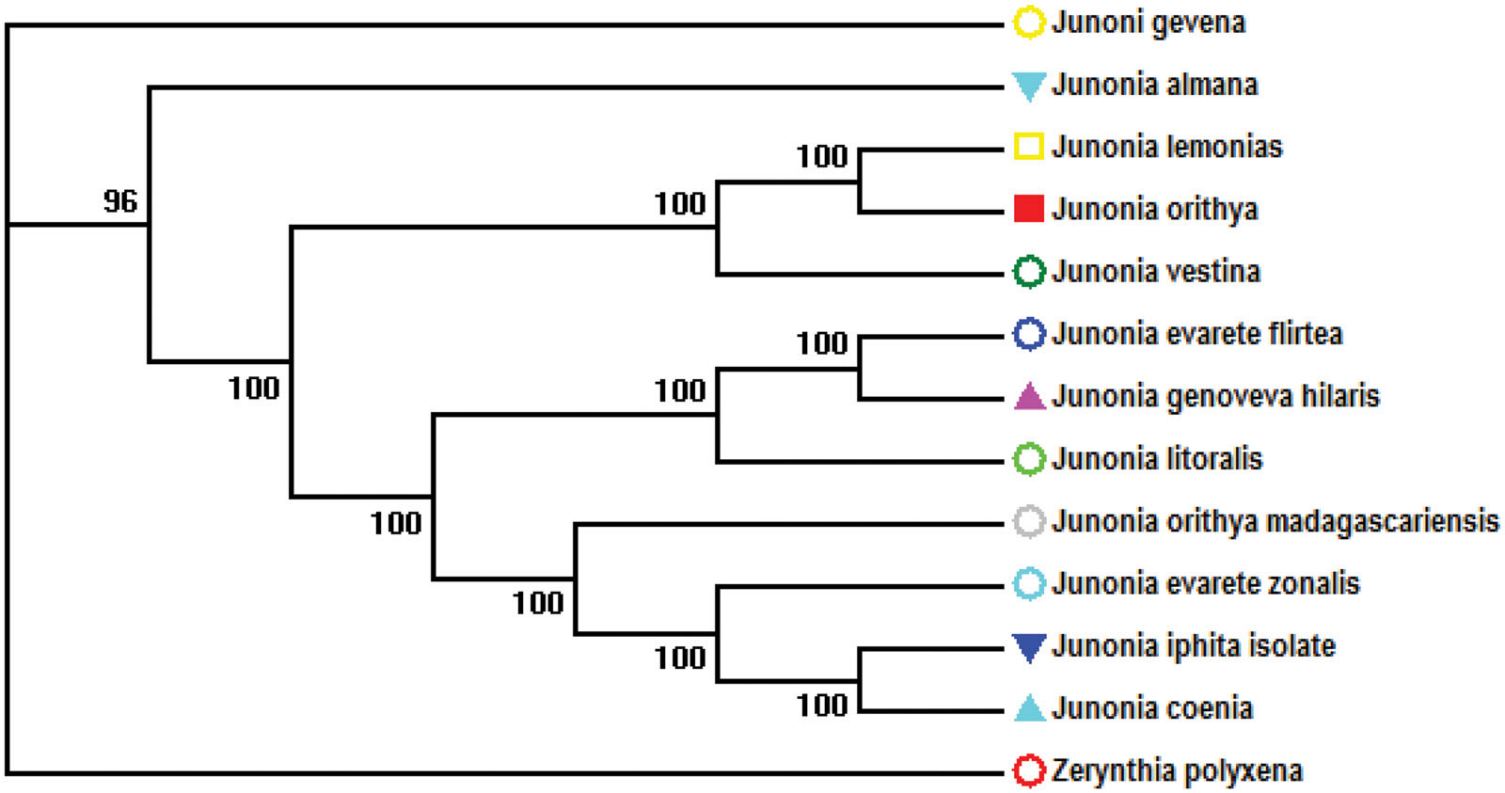
Evolutionary relationships of taxa. The evolutionary history was inferred using the Neighbor-Joining. The evolutionary distances were computed using the Maximum Composite Likelihood method. There were a total of 14,908 positions in the final dataset. Evolutionary analyses were conducted in MEGA6.gene bank accession numbers *Junonia orithya* has been activated (KF199862.1), retrived sequences Accestion numbers *Junonia lemonias* (KX423731.1), *Junonia vestina* (KX423728.1), *Junonia coenia* (KX267579.1, KX267570.1), *Junonia genoveva* (KX267570.1), *Junonia genoveva hilaris* (KX267570.1), *Junonia orithya madagascariensis* (KX267581.1), *Zerynthia polyxena* (MK507888.1), *Junonia iphita* isolate (KU577289.1), *Junonia almana* (KF590539.1), *Junonia litoralis* (KX267568.1), *Junonia evarete zonalis* (KX267573.1).

## Data Availability

The authors confirm that the data supporting the findings of this study are available within the article [and/or] its supplementary materials and available in https://www.ncbi.nlm.nih.gov/ under Accession: KF199862.1 GI: 1559463072.
